# A definitive randomised controlled trial of a group mindfulness-based psychological therapy for people with psychosis: the MIND-P study

**DOI:** 10.1186/s12888-025-07470-3

**Published:** 2025-11-26

**Authors:** Lyn Ellett, Laura Dannahy, Kate Bennett, Maria Chorozoglou, Beth Ford, Zachary Howarth, Joshua Phillip Waterman, Adedamola Falana, Katherine Berry

**Affiliations:** 1 School of Psychology, University of Southampton, Southampton, UK; 2 Hampshire and Isle of Wight Healthcare NHS Foundation Trust, Southampton, UK; 3 Barts Clinical Trials Unit, Queen Mary University of London, London, UK; 4 Greater Manchester Mental Health NHS Foundation Trust, Manchester, UK; 5 University of Manchester, Manchester, UK

**Keywords:** Mindfulness, Psychosis, Schizophrenia, Group therapy, Randomised controlled trial

## Abstract

**Background:**

Schizophrenia is a severe mental illness with a significant disease, economic and social burden. Persecutory delusions (beliefs that one will be harmed or mistreated) are a common symptom of schizophrenia with associated high levels of depression. International clinical guidelines recommend individual cognitive behaviour therapy for schizophrenia, but there are currently no group psychological therapies recommended in NICE guidelines. We conducted a pilot randomised controlled trial, comparing group mindfulness therapy (GMT) to treatment as usual (TAU) for people with schizophrenia and persecutory delusions where we demonstrated feasibility and acceptability. It is now timely to progress to conduct a definitive randomised controlled trial (RCT).

**Methods:**

A parallel group RCT with single blind assessment comparing GMT + TAU (intervention condition) with TAU alone (control condition). The PHQ-9 and additional patient-reported psychometric measures will be collected at three time points: at baseline (prior to randomisation), end of therapy (approx 4 months post randomisation), and follow up (approx 8 months post-randomisation). Participants will be 144 adults with a schizophrenia spectrum diagnosis with current persecutory delusions or attending an early intervention in psychosis service, recruited across 3 NHS sites (Hampshire and Isle of Wight Healthcare NHS Foundation Trust, Greater Manchester Mental Health NHS Foundation Trust, and Pennine Care NHS Foundation Trust), randomised 1:1 to intervention or control groups. The therapy will be delivered according to our published manualised protocol. We will also monitor and evaluate a range of safety indices throughout the trial, conduct mediation and moderation analyses to understand how, and for whom, the therapy is most effective and assess cost effectiveness.

**Discussion:**

If shown to be effective and cost-effective, GMT will positively impact the lives of people with schizophrenia, by providing an evidence-based group therapy. This is particularly important as there are no group psychological therapies for schizophrenia in the NICE guidelines and group therapies offer the potential for cost savings for service providers. The findings will be disseminated to a range of stakeholders, including service users/carers, academic researchers, clinicians, research participants and the general public, via academic publications, conferences, plain English summary (written and video versions) and public engagement events.

**Trial registration:**

ISRCTN: ISRCTN16318074, registered on 07/02/2025.

**Supplementary Information:**

The online version contains supplementary material available at 10.1186/s12888-025-07470-3.

## Background

Schizophrenia is a severe mental illness that affects how a person thinks, feels and behaves, and has been referred to as the “abandoned illness” [1]. Worldwide, 24 million people are currently diagnosed with schizophrenia [2], and in the UK, it is over 650,000 people (around 1% of the population) [3]. The burden of schizophrenia is substantial, with impacts including earlier mortality, multiple psychiatric comorbidities such as depression, poor social functioning and unemployment [4]. People with severe mental illness, including schizophrenia, are at risk of dying on average 15 to 20 years earlier than other people. In addition to the disease burden for individuals themselves, schizophrenia also has the highest median societal cost per patient of all mental disorders [4]. The economic cost of treating schizophrenia globally is also very high – for example, mental illness costs the UK economy in excess of £77 billion per year [5], and schizophrenia accounts for around 30% of all spending in adult mental health in the NHS [6]. In the US, the societal cost of schizophrenia was reported as US$281.6 billion in 2022 [7]. Finally, in a recent systematic review of cost-of-illness studies (*k* = 24), the annual societal cost of schizophrenia per person was US$819 in Nigeria to US$94,587 in Norway [8].

One of the most common and disabling symptoms of schizophrenia is persecutory delusions, which occur when individuals believe that other people are intentionally trying to mistreat, harm or kill them [9]. Almost half of individuals with persecutory delusions have levels of well-being in the lowest 2% of the general population [10] and depression has been shown to be high in this population and predicts the persistence of persecutory delusions [11]. Given the high social, economic and individual costs of schizophrenia, and the impact of persecutory delusions in particular, it is important to improve the range of evidence-based treatments available, including seeking new ways to help individuals suffering from this debilitating mental health condition.

One of the first line treatments for schizophrenia recommended internationally in clinical guidelines is Cognitive Behaviour Therapy for psychosis (CBTp), including, for example in England and Wales, Germany, Canada, Australia and New Zealand [6, 12–14]. CBTp typically targets individual psychotic symptoms, including delusions, voices and paranoia, and is typically delivered in an individual format. There is, however, a gap in the evidence base and in current healthcare provision, as there are currently no NICE-recommended group psychological therapies for people with schizophrenia. This is important because group therapies offer the potential (subject to full health economic analyses) for cost savings for healthcare providers.

Mindfulness-based interventions (MBIs) are increasingly being used to help people with schizophrenia and are frequently delivered in a group format [15],and the evidence base for MBIs for schizophrenia is growing [16]. Mindfulness-based approaches might be particularly helpful for people with persecutory delusions as mindfulness offers the opportunity to ease distress without focussing specifically on the content of persecutory beliefs [17, 18]. A recent meta-analysis summarising the effects of group mindfulness therapy for people with schizophrenia compared with treatment as usual (*k* = 4 studies), reports an overall medium effect size (Hedge’s g = 0.46) on symptom reduction [19], but the review also concluded that there is currently no data or evidence for people with schizophrenia who experience persecutory delusions. The overall range of effect sizes reported in a recent summary of 10 published meta-analyses was 0.45–0.86, with small effects reported in three reviews, medium effects in six reviews and large effects in one review [16]. In recognition of the absence of evidence of MBIs specifically for people with persecutory delusions, and the importance of addressing this evidence gap, we conducted a pilot RCT, comparing group mindfulness alongside treatment as usual with treatment as usual alone for people with schizophrenia and persecutory delusions [20]. In our pilot trial (*n* = 27), we demonstrated feasibility and acceptability (of conducing an RCT in this patient group, of participation in a randomised controlled trial, and of the intervention itself): (1) Recruitment was completed within the pre-set timeframe; (2) 96% of participants were retained in the study (only 1 person dropped out from the treatment as usual only arm); (3) All participants allocated to receive group mindfulness therapy completed it, demonstrating acceptability of the intervention; (4) 100% adherence to the therapy protocol was achieved by all trial therapists; (5) We were able to show a signal of effect on the primary outcome (Cohen's d = 0.2); and (6) 64% who received the therapy showed a clinically significant reduction in depression symptoms. Given the success of the pilot, we have now progressed to a definitive trial to evaluate the clinical and cost effectiveness of GMT for people with schizophrenia and persecutory delusions.

As well as understanding the beneficial effects of any intervention, it is also important to evaluate any potential harmful effects. The importance of understanding any potential harmful effects of mindfulness is a current live issue in the field [21]. We recently published international guidelines for operationalising and monitoring harm in randomised controlled trials of mindfulness therapies for people with schizophrenia [22]. Using these guidelines, this current trial will evaluate safety indices in a definitive clinical trial of mindfulness for schizophrenia.

The overall aim of the study is to assess the effectiveness and cost effectiveness of GMT for people with schizophrenia and persecutory delusions. The study has the following objectives: (1) assess the effectiveness of GMT by conducting a definitive RCT comparing GMT + TAU vs TAU (TAU was chosen as the comparator as this is the first definitive trial of group mindfulness for people with persecutory delusions, and therefore TAU was considered the most appropriate control condition); (2) conduct a full health economic evaluation to determine the cost effectiveness of GMT; (3) assess adherence to the therapy protocol; (4) evaluate how GMT works and for whom the therapy is most effective; (5) undertake a full evaluation of safety indices in a definitive RCT of GMT.

Primary hypothesis: Compared with TAU, GMT + TAU will result in a significant reduction in depression immediately after the intervention (4 months post randomisation).

Secondary hypothesis 1: Compared with TAU, GMT + TAU will result in a significant reduction in depression at follow up (8 months post randomisation).

Secondary hypothesis 2: Compared with TAU, GMT + TAU will result in greater progress towards recovery, will reduce loneliness and will increase forgiveness, both after the intervention (4 months post randomisation), and effects will be maintained at follow up (8 months post randomisation).

Exploratory hypothesis: Compared to TAU, TAU + GMT will improve mindfulness and anxiety, which will mediate clinical outcomes. We will test potential moderators of effects, including clinical variables (illness duration, baseline symptom severity) and demographics (age, gender) to identify patients who may benefit more or less from GMT.

Exploratory research question: Will there be a difference in safety indices (number of serious adverse events and adverse events, drop out, hospital admissions, symptom deterioration) between TAU and GMT + TAU groups?

## Methods/design

### Study design

The design is a multi-centre, parallel group definitive randomised controlled trial with single blind assessment comparing GMT + TAU (intervention condition) with TAU alone (control condition). TAU will be measured and remain as usual in both groups. Assessments will be carried out at baseline, post therapy (4 months post randomisation) and follow up (8 months post randomisation) by study research assistants. This trial is registered at ISRCTN (ISRCTN16318074) and complies with the World Health Organization Trial Registration Data Set. Any changes or modifications to the study protocol (e.g., adding questionnaires, altering study procedures) will be formally submitted to the HRA for approval. The study may be subject to an audit at any time either by Queen Mary University of London Barts Clinical Trials Unit, the study sponsor (University of Southampton) or the Health Research Authority. This study is designed as a randomised controlled trial and will be reported in accordance with the CONSORT (Consolidated Standards of Reporting Trials) 2010 guidelines. The trial protocol, at the time of submission, is Version 6 (17.12.2024). The trial is funded by the National Institute for Health Research (NIHR), Research for Patient Benefit Programme (NIHR206786).

### Sample size

To determine a clinically significant difference between conditions at post treatment of 5 points on our primary outcome depression (consistent with pilot data), with 90% power and a significance level of 0.05, would require 120 evaluable participants. Consistent with the pilot data, the calculation assumes a standard deviation of 14 in depression, and a correlation between pre- and post- depression scores of 0.8. To allow for attrition of up to 15% (based on average attrition reported in the literature), we will aim to recruit 144 participants.

### Randomisation

Randomisation will be managed by Queen Mary University of London Barts Clinical Trials Unit (BCTU). Participants will be randomised 1:1 to either the intervention group (mindfulness therapy + TAU) or control group (TAU alone). Randomisation will be stratified by NHS site, sex and cohort, using a minimisation algorithm incorporating a random element to ensure balance across minimisation factors and concealment of the randomisation allocation. Unblinded research personnel at each site will be informed of the randomisation allocation by the statistician at BCTU and will inform the patient of their allocation. The trial team research staff involved in data capture will remain blinded to the treatment allocation. Eligibility and consent will be verified before each patient is randomised.

### Participants and recruitment

Participants will be 144 adults, recruited across three sites – Hampshire and Isle of Wight Healthcare NHS Foundation Trust (HIoW), Greater Manchester Mental Health NHS Foundation Trust (GMMHT) and Pennine Care NHS Foundation Trust. Participants will primarily be recruited through secondary care community mental health teams (CMHTs) and Early Intervention in Psychosis teams. Recruitment is expected to be completed within 18 months of the start of the trial (March 2026).

### Inclusion criteria

1. Adult aged 18 or over; 2.Current diagnosis of Schizophrenia, Schizoaffective Disorder, Delusional Disorder or Psychosis NOS (confirmed by multidisciplinary team), or attending an early intervention in psychosis service; 3.Presence of a current persecutory delusion (assessed using the delusion subscale of the PSYRATS at baseline with a score of at least 2 on item 4 or 5, consistent with previous research).

### Exclusion criteria

**Fig. 1:**
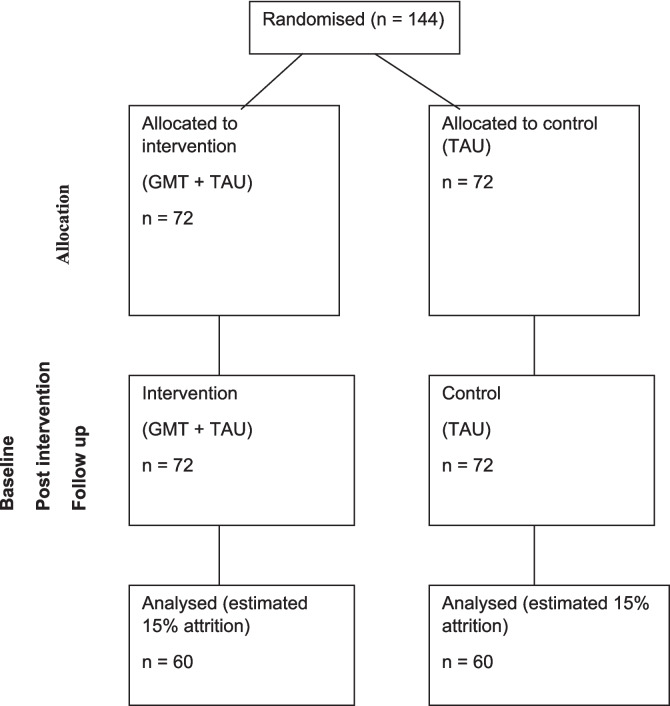
CONSORT diagram showing flow of participants

1. Primary diagnosis of substance dependency or personality disorder; 2. Identified organic syndrome or moderate/severe learning disability; 3. Active engagement in any other individual or group-based therapy at the time of entry into the study (note study participants will not be denied access to any therapy offered as part of TAU during the study); 4. Unable to give informed consent. Figure 1 shows anticipated participant flow through the study.

### Consent

The study research assistants will seek written informed consent if meeting the participant face-to-face, or verbal informed consent if contact occurs remotely. If verbal consent is sought, the researcher will keep a log of the date that informed consent was given. The research assistant will audio-record informed consent via a trust approved video conferencing system or by telephone.

## Study arms

### Group mindfulness therapy (GMT)

Our research team developed and published a manualised group mindfulness therapy protocol [15], which we used in our pilot trial with people with persecutory delusions [20]. Mindfulness group therapy will be conducted over 10 group sessions, each lasting 1.5 h. Mindfulness will be practiced at all 10 sessions, and home practice will be encouraged and supported through the provision of audio files and equipment if needed. Each therapy group will be run by two therapists (Clinical Psychologists or CBT practitioners), and weekly supervision will be provided by a Clinical Psychologist to the therapists.

### Treatment as usual (TAU)

TAU will be the usual treatment offered in clinical services in the UK for people with schizophrenia (e.g. community mental health teams or early intervention in psychosis teams), and typically involves psychiatric consultation, medication, and key worker support. The number of contacts with psychiatrists and keyworkers for each participant will be recorded from electronic patient records, to adequately describe ‘TAU’ and ensure comparability across sites.

### Assessment of adherence to therapy protocol

To assess adherence to therapy protocol, we will use a Therapy Session Checklist, which has been used in previous studies [23]. The therapy session checklist will be administered by the unblinded researcher at 3 randomly chosen therapy sessions for each therapy group, and percentage adherence will be reported.

#### Clinical outcomes

- Primary Outcome: Depression measured using the Patient Health Questionnaire 9 (PHQ-9). This is a 9-item measure of depression, with excellent internal consistency (α = 0.83), and is commonly used in routine clinical practice [24].
- Justification for primary outcome: Depression was identified as the primary outcome for the trial with the following justification: 1. Depression is associated with persecutory delusions, is highly comorbid in people with schizophrenia and has been shown to predict persecutory delusion persistence in a longitudinal study [11]; 2. Depression has been identified by experts in the field as one of the most important clinical outcomes for this client group when evaluating psychological therapies; 3. Depression was recommended as the primary outcome by our PPIEP group and was the primary outcome in our pilot trial.

We will also measure the following clinical outcomes:

Severity of psychotic symptoms, including delusions and voices, will be measured using the Psychotic Symptoms Rating Scales (PSYRATS) [25].

Recovery will be measured using the Recovery Assessment Scale (RAS). This is a 24-item measure of personal recovery, with excellent internal consistency (α = 0.92). It is one of the most widely used measures of personal recovery [26].

Forgiveness will be measured using the 10-item Trait Forgivingness Scale, which has excellent internal consistency (α = 0.92) and loneliness will be measured using the 3-item UCLA Loneliness Scale which has good internal consistency (α = 0.81) [27].

#### Mediators

- Mindfulness skills: Southampton Mindfulness Questionnaire (SMQ). This is a 16-item questionnaire that assesses mindfulness skills, designed specifically for people with schizophrenia, with excellent internal consistency (α = 0.83) [28].
- Anxiety: Generalised Anxiety Disorder Questionnaire (GAD-7). This is a 7-item measure of anxiety, with excellent internal consistency (α = 0.92), and is commonly used in routine clinical practice [29].

#### Moderators

Demographic characteristics (age, gender), illness duration, baseline symptom severity.

#### Health economic outcomes

To assess health-related quality of life, we will use the EQ-5D-5L [30], one of the most commonly used generic measures of health for clinical and economic appraisal, with good internal consistency (α = 0.79), and the Recovering Quality of Life Scale (ReQoL-10), a 10-item measure of mental health, also with excellent internal consistency (α = 0.88) [31]. We will also collect and report data on secondary care resource use using the Patient Level Information and Costing System in the NHS (PLICs) and the Client Service Receipt Inventory (CRSI) [32].

### Data management

Queen Mary University of London Barts CTU will oversee data management. Study data will be collected and managed using REDCap electronic data capture tools hosted at BCTU QMUL (Trial-specific case report forms will be developed and used for data collection). To maintain patient confidentiality, all identifiers will be removed, and only anonymised data will be entered. A full data management plan will be prepared by the data manager at BCTU and reviewed by the statistician before the onset of data collection. This will detail the processes for data management that will be adhered to throughout the trial. Data access requests will be considered by the chief investigator, at the end of the trial and following publication of the main findings. Any requests will need to be accompanied by a summary of the proposed study for which the data is sought and a data sharing agreement. All participant data will be held on password protected computers or in locked filing cabinets at the University of Southampton or at participating NHS Trust sites. Consent forms and any other identifiable information will be kept in a separate locked filing cabinet or computer file. Access to data will be limited to members of the research team and relevant regulatory authorities. Trust and University Governance procedures will be adhered to throughout the full duration of the trial.

### Withdrawal criteria

Participants are free to withdraw from the study at any time without any negative consequences to themselves, but the type of withdrawal will be categorised, for example withdrawal from the study (prior to randomisation), treatment, withdrawal from further follow-up. All participants will be encouraged to complete follow-up to maximise data capture, even if they have withdrawn from treatment, but their rights to withdraw from all further participation in the trial will be respected. All data collected up until the date of withdrawal will be included in the final analysis under the intention to treat principles, unless a participant explicitly withdraws consent for the use of any data collected. The study may be terminated prematurely in the case of any serious adverse event directly linked to trial participation, but this will be discussed by the TSC.

### Statistical analysis plan

A detailed Statistical Analysis Plan (SAP) will be developed and finalised prior to database lock and before any unblinded data are accessed by the study team or statisticians involved in the analysis. The SAP will outline the analysis populations, primary and secondary outcome analyses, covariate adjustment, handling of missing data, model specifications, subgroup analyses, and any planned sensitivity analyses. The final SAP will be signed off by the study statistician and relevant members of the study team and will guide all prespecified analyses for the trial. The analysis of all validated questionnaires will use the relevant published scoring guidelines, the details of which will be included in the SAP.

### Descriptive summaries

The study flow and participant characteristics by randomised group will be reported. Responses to patient reported outcome measures will be summarised by randomised group at baseline, 4-months and 8-months. Summary measures for continuous measures will be mean and standard deviation, or median and interquartile range.Categorical data will be presented as number and percentage.

### Primary outcome analysis

Primary analyses will use a mixed model approach, comparing symptom scores at 4- and 8-months post-randomisation between groups, adjusting for baseline symptoms and other pre-specified factors as fixed effects, and including a time by group interaction effect as part of the fixed effects specification. The primary outcome will be determined at four weeks post-randomisation. Random participant and cohort effects will account for repeated measurements within subjects and cohort groups. All analyses will be undertaken according to the intention to treat principle, analysing participants according to their randomised treatment allocation regardless of their adherence to the treatment protocol. The primary outcome will be presented as adjusted coefficient and 95% confidence interval.

### Secondary and exploratory outcome analyses

Secondary symptom score outcomes will be analysed as for the primary outcome. Secondary reporting of model estimated effects at 8-weeks post-randomisation will be presented.

#### Exploratory analyses: mediators and moderators

To explore possible mechanisms that explain the differences between the intervention and control groups, mediation analyses will examine if the intervention works through the pathways of increasing mindfulness and reducing anxiety. To gather information on intervention tailoring, moderator analyses will explore the interaction between clinical variables (illness duration, baseline symptom severity) and demographics (e.g., age, gender). These exploratory analyses will also be conducted in a linear mixed model or generalized linear mixed model framework. Mediation will be examined by adding the proposed mediators to the model and calculating the extent to which the main treatment effect changes between the main effect and mediation models. Post-hoc calculations can be used to describe the proportion of the main treatment effect mediated (and confidence interval). We will consider possible mediators separately and jointly. Moderation will be assessed by adding the hypothesized moderator to the main analysis model. We consider these tests exploratory and to be used to inform future trials.

#### Safety indices

In relation to safety indices that will be monitored throughout the trial: We will report descriptive statistics for safety indices (number of serious and adverse events, drop out, hospital admissions, symptom deterioration) for each condition (GMT + TAU vs TAU alone) during the trial, and report any differences in each safety index by trial arm using an appropriate analysis method.

#### Health economic evaluation

We will conduct a full economic evaluation assessing the cost effectiveness of the proposed intervention as compared to TAU. We will collect and report data on secondary care resource use using PLICs (Patient Level Information and Costing Systems) data. We will also collect data on HRQoL using the Euroqol EQ-5D-5L recommended by NICE [32] (NICE., 2019) and the Recovering Quality of Life (ReQoL-10, Keetharuth et al., 2018) for mental health, which is used to assess the recovery process of mental health conditions. We will estimate the cost of providing the protocolised intervention to assess the impact to the NHS. Health economic data will be recorded at the same time points as the clinical data. The within trial economic analysis, adherent to guidelines for good economic evaluation [33, 34], will include (i) cost-effectiveness analysis (CEA) using the primary outcome (depression), and (ii) cost-utility analysis (CUA) using HRQoL (EQ-5D-5L) data. Sensitivity analysis will explore potential differences occurring from the two QoL instruments. Resource use will be assessed using PLICS data and the CRSI [35].

The perspective of the economic sub-study will be that of the NHS. All cost-effectiveness results will be presented on: (i) the cost-effectiveness plane, which captures the uncertainty around the results showing the incremental costs and incremental effects of the comparison of interest in a 2-dimentional plot, and (ii) the cost-effectiveness acceptability curves, which graphically represent the uncertainty in terms of probabilities, regarding the cost effectiveness of the intervention.

## Discussion

The overall aim of the study is to conduct a definitive RCT to assess the effectiveness and cost effectiveness of GMT for people with schizophrenia and persecutory delusions (or those attending an EIP service where they may not necessarily have a diagnosis).

### PPIEP

PPIEP has been central to the design and development of the study from the outset. We used INVOLVE guidelines and the Public Involvement Impact Assessment Framework to develop our PPIEP plans. PPIEP has contributed to the study so far by: (1) establishing the importance and need for the study; (2) confirming acceptability of the randomisation element of the study; (3) identifying important outcomes for service users, including contributing to identifying the primary outcome for the trial; (4) ensuring the number of outcome measures is not overly burdensome; (5) contributing to the Plain English Summary for the funding application; (6) advising on support needed for individuals not randomised to receive the therapy and consideration of barriers to recruitment and engagement to develop and inform our recruitment strategy.

There will be PPIEP in all elements of the research project lifecycle throughout the full duration of the research, including: (1) management of the research as members of the TMG, with representatives attending both the TMG and TSC meetings; (2) on the interview panels for recruitment of the two study research assistants; (3) collaborating in developing participant resources and advising on ways to maximise recruiting participants into the trial; (4) coproduction of the participant feedback survey; (5) advising on safety indices to monitor and report on throughout the trial; (6) contributing to study reporting and dissemination, by co-producing the lay summary of findings, co-producing the video and co-presenting findings. We also plan to co-produce a paper for publication to disseminate our PPIEP activities and their impact, which will be led by our PPIEP group.

### Governance and trial management

The trial sponsor is the University of Southampton. The Trial Management Group (TMG) includes the two joint lead applicants, site PIs , PPIEP lead ,the study research assistants, the trial statistician, and the data manager. The TMG meets monthly throughout the trial, and will have responsibility for the set-up, running and analysis of the research. The Trial Steering Committee (TSC) will have overall oversight of the trial and will provide advice to the trial management group, funder and sponsor of the research when required. The TSC has an independent chair, PPIEP representative and statistician. Given the size of the trial, a separate DMC has not been convened. The functions typically carried out by a trial DMC, such as monitoring patient safety and the integrity of the data, will be overseen by the TSC.

### Challenges

We are aware of the specific challenges associated with designing, planning and implementing clinical trials evaluating group interventions, which have been recently documented in the literature [36]. For example, group intervention trials involve a delay in starting the intervention for some participants, until a sufficient number of participants have consented to take part, have been randomised and are then available to start a group. We will address this by ensuring that our unblinded study research assistants communicate regularly with consented participants to keep them updated about timings and likely group start times. The main barrier to the proposed work is likely to be difficulties and delays with recruitment of participants into the study, and therefore recruitment is key to success. We have developed a recruitment strategy with our PPIEP group, which we will collectively monitor throughout the trial.

### Dissemination

Our dissemination plan includes: (a) academic publications in peer-reviewed journals reporting the main trial findings (effectiveness and cost effectiveness) and PPIEP activities and impact; (b) conference presentations; (c) a written and video version of the plain English summary of findings, co-produced with our PPIEP group; (d) dissemination through UK and international specialist academic centres.

## Supplementary Information

Supplementary Material 1.

## Data Availability

The study materials and datasets used and/or analysed during the current study are available from the corresponding author on reasonable request.
